# The effects of high versus low frequency of combined physical and cognitive training on cognitive function in older adults with cognitive decline: a quasi-experimental study

**DOI:** 10.1186/s12877-023-03802-8

**Published:** 2023-02-14

**Authors:** I-Ching Chuang, I-Chen Chen, Ken-Hsien Su, Yih-Ru Wu, Ching-Yi Wu

**Affiliations:** 1 Department of Occupational Therapy and Graduate Institute of Behavioral Sciences, College of Medicine, Chang Gung University, No. 259 Wen-Hwa 1St Road, Taoyuan, Taiwan; 2 Department of Neurology, Linkou Chang Gung Memorial Hospital, Taoyuan, Taiwan; 3 Department of Occupational Therapy, College of Nursing and Health Sciences, Da-Yeh University, Changhua, Taiwan; 4 College of Medicine, Chang Gung University, Taoyuan, Taiwan; 5 Healthy Aging Research Center, Chang Gung University, Taoyuan, Taiwan; 6 Department of Physical Medicine and Rehabilitation, Linkou Chang Gung Memorial Hospital, Taoyuan, Taiwan

**Keywords:** Frequency, Cognitive decline, Physical exercise, Cognitive training

## Abstract

**Background:**

The effects of combined training can be affected by training characteristics such as frequency, session length, and duration. No empirical studies to date have directly compared how combined physical and cognitive training offered at different training frequencies affects cognitive function for older adults with cognitive decline. This study investigated the impact of training frequency on cognitive outcomes after combined physical and cognitive training for older adults with cognitive decline.

**Methods:**

A quasi-experimental study was conducted in community facilities and day care centers. The study assigned 89 older adults with cognitive decline into high-frequency (HF) or low-frequency (LF) training groups. The participants received 90- to 105-min training sessions, one (LF) or three (HF) times a week, for 12 weeks. Outcome measures were the Montreal Cognitive Assessment, Word List subtest of the Wechsler Memory Scale, Digit Symbol Substitution Test (DSST), and Stroop Color Word Test.

**Results:**

The HF group demonstrated greater improvement in immediate memory measured by the WL-IM (*F* = 8.7, *P* = 0.004) and in executive function measured by the SCWT (*F* = 5.89, *P* = 0.017) than the LF group. Compared with the HF group, the LF group showed a great improvement in delayed memory measured by the WL-DM (*F* = 9.62, *P* = 0.003). The HF and LF groups both increased in processing speed and global cognitive function.

**Conclusions:**

Our study indicated that the different training frequency of combined physical and cognitive training may result in benefits on different cognitive functions in older adults with cognitive decline. These findings may assist clinical practitioners in choosing appropriate training frequencies based on various intervention purposes for the elderly with cognitive decline.

**Trial registration:**

ClinicalTrials.gov Identifier NCT03619577 (08/08/2018).

## Introduction

With a rapidly growing aging population, the incidence of cognitive decline, such as mild cognitive impairment and dementia, is increasing [1]. Cognitive decline may interfere with independence in everyday activities [2], reduce quality of life, and contribute to health care costs [3]. Providing effective interventions to alleviate and prevent cognitive decline is necessary.

Accumulating evidence indicates that physical exercise combined with cognitive training may improve cognitive function for older adults with cognitive decline [4–6]. Physical exercise can help create the conditions for neuroplasticity to occur, and cognitive training can support these processes by providing the brain with new and challenging experiences that stimulate neural activity and promote changes in the brain [7]. Studies have shown positive effects of combined training on global cognition, short-term and working memory, and executive function for older adults with cognitive decline [5, 6]. However, the effects of combined training can be affected by training characteristics such as frequency, session length, and duration [6, 8]. For developing an appropriate program and optimizing training effectiveness, investigating how training characteristics influence the effects of combined training is critical.

Zhu et al. (2016) investigated how the training characteristics of combined cognitive and physical training influenced cognitive performance for community-based older adults. They investigated the effects of different session length (≤ 1 h vs. > 1 h), duration (≥ 16 weeks vs. < 16 weeks), and training frequency (< 5 sessions/week vs. ≥ 5 sessions/week) on cognitive function. They found that the session length and duration did not affect cognitive performance; however, the training frequency did influence the cognitive effects of combined training [8].

Systematic reviews and meta-analyses of effects of combined cognitive and physical training have reported analyses of frequency impacts. These review studies reported that older adults who received a lower frequency (1–2 sessions/week) of combined training had better cognitive performance, denoted by the magnitude of the effect size, than those who received a higher frequency (3–5 sessions/week) [8, 9]. The possible reason might be that the combination of two elements—exercise and cognitive training—exerted intense and diverse demands that led to cognitive fatigue and excessive stress [8–10] in those who received the higher frequency of training.

The training frequency may affect cognitive skill acquisition [11, 12]. Low-training frequency may lead to better learning outcomes than high-training frequency (eg, spacing effect) [11, 13]. The low-frequency schedule may be related to the survival of new neurons in the hippocampus and cause more neural changes [14]. However, the intense schedule may give rise to more stored information for a short period of time, but not neural changes, which are essential for training transfer [13].

Contradictory to the previous review studies and skill acquisition theory discussed above, Gheysen et al. (2018), conducting the potential moderator analysis of the efficacy of a combined physical and cognitive intervention, found frequency (≥ 3 vs. 1 session/week) did not significantly influence the effects of combined training on cognition. Although the findings of Gheysen et al. did not favor low-frequency training, there was a trend that the effect size in the low-frequency training was higher than in the high-frequency training. High-frequency training might lead to more effective short-term learning than low-frequency training [15] and produce benefits in short-term memory [16]. These discrepancies may arise within the different cognitive outcomes that are measured.

No empirical studies to date have directly compared how different frequencies of combined physical and cognitive training affect cognitive function for older adults with cognitive decline. It is critical to search for the precise amount of training that is sufficient, but not overwhelming, to alleviate cognitive decline and enhance cognitive function for older adults with cognitive decline. This study investigated the impact of training frequency on cognitive outcomes after combined physical and cognitive training for older adults with cognitive decline. We hypothesized that combined physical and cognitive training at different frequencies may lead to benefits on different cognitive functions in older adults with cognitive decline.

## Method

### Participants

The local ethics committee and institutional review board approved the study protocol. Participants were recruited from local community facilities and day care centers. The inclusion criteria were (1) age ≥ 60 years, (2) having self- or informant-reported cognitive complaints, (3) the ability to follow instructions (≥ 17 points on the Mini-Mental State Examination), (4) < 26 points on the Montreal Cognitive Assessment (MoCA), (5) no difficulty with basic activities of daily living, and (6) no diagnosis with dementia by a neurologist. Participants were excluded if they had recent myocardial infarction, heart failure, recent heart surgery, severe asthma, cognitive decline concomitant with other neurologic disorders, or an unstable medical condition that might prevent them from performing physical exercise or cognitive training.

### Study design and procedure

**Fig. 1:**
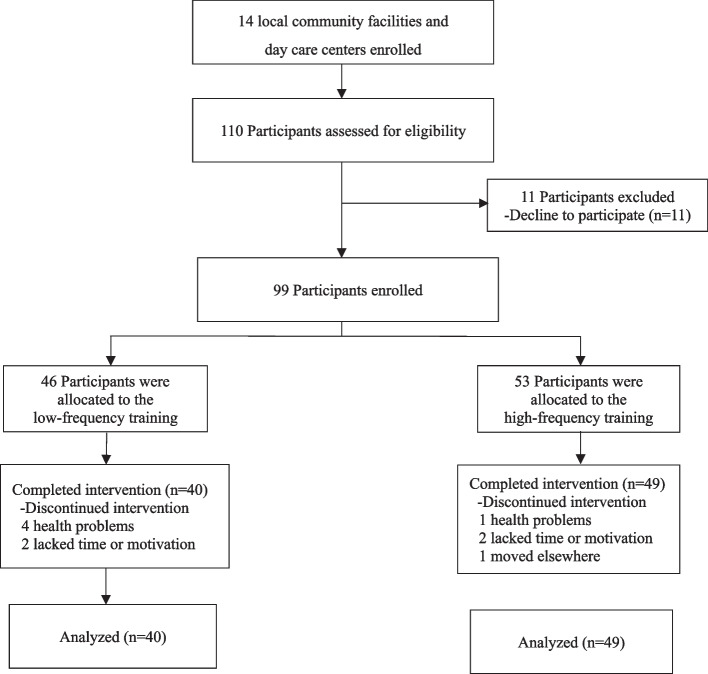
Flowchart of participant disposition throughout the study

This study was a multicenter and controlled trial. Fourteen local community facilities and day care centers in northern Taiwan were enrolled between 2018 and 2021. Participants engaged in high-frequency (HF group) or low-frequency (LF group) sessions of combined physical and cognitive training. For the HF group, we chose a small number of sessions (3 sessions/week) to avoid fatigue and excessive stress caused by high-frequency training [9]. Similarly, we also chose the minimal number of sessions (1 session/week) for the LF group. We determined from previous studies that combined physical exercise and cognitive training for 90 to 120 min per session for at least 12 weeks seems to lead to significant improvements in cognitive performance [17, 18]. Thus, training in the HF group consisted of a 90- to 105 min session three times weekly for 12 weeks, whereas training in the LF group consisted of a 90- to 105-min session once weekly for 12 weeks. Over the course of the study, 89 participants completed all of the training sessions (Fig. 1).

### Intervention

Training sessions were conducted in groups of 5 to 10 participants, led by a certified therapist. Participants engaged in physical exercise for 45 to 55 min, followed by 45 to 50 min of cognitive training. The participants performed 10 min of warm-up, followed by 30 to 35 min of physical exercise, and ended with 5 to 10 min of cool-down. The physical exercise programs involved aerobic exercises, resistance training, and balance training. For aerobic exercise, such as stepping and walking, the target heart rate was set at 40% to 70% of the maximal heart rate. Equipment for resistance and balance training included water bottles, wrist or ankle weights, Thera-Band, and exercise balls. The therapist increased the physical exercise intensity as the participants improved their performance throughout practice.

After physical exercise training, the participants took part in 45 to 50 min of cognitive training. In addition to using a computerized cognitive program, BrainHQ (Posit Science Inc., San Francisco, CA, USA) for cognitive training, we designed a PowerPoint presentation with content derived from BrainHQ and incorporating the functional elements. The domains of cognitive training consisted of attention, memory, processing speed, visuospatial skill, and calculation. Participants practiced two or three cognitive domains in each training session. In the memory task, for example, participants were asked to memorize a series of numbers and then say them backward. Each session contained 10 min of warm-up (explaining the task rules and paying attention to the therapist), 30 to 35 min of cognitive training, and 5 min of cool-down (providing home programs and feedback to the tasks). The tasks were adjusted continuously based on the participant’s level of performance.

### Outcome measures

The participants were assessed before and immediately after the training programs. The assessors were masked to the group of participants when they conducted the evaluation. We used reliable and valid assessments, including the Montreal Cognitive Assessment (MoCA), Digit Symbol Substitution Test (DSST) of the Wechsler Adult Intelligence Scale (WAIS), Word List (WL) of the Wechsler Memory Scale–third edition (WMS-III), and the Stroop Color Word Test (SCWT), to assess cognition in older adults.

The MoCA is a widely used instrument to evaluate global cognitive function and has good sensitivity for detecting cognitive decline [19]. Global cognitive functioning refers to a family of cognitive capacities, including orientation, attention, memory, naming, visuospatial, executive function, language, and abstraction. The total score is 30 points, and higher scores represent better global cognitive function.

The DSST, a subtest of WAIS, was used to assess information processing speed [20]. The participant is asked to match symbols to numbers and copy the symbols into spaces within 120 s. The score of DSST is the number of correct symbols. The DSST is sensitive to detect cognitive impairment and change of cognitive function [21]. Higher scores on the DSST indicate better information processing speed.

The WL is a subtest of WMS-III that is the commonly used battery for testing working memory [22]. The WL test includes two parts, WL-I and WL-II. In WL-I, 12 unrelated words are presented in four trials, and the participant is asked to recall the words immediately after each presentation. For WL-II, 25 to 35 min after completing the WL-I, participant is asked to again recall the 12 unrelated words presented earlier. The WL-Immediate Memory (WL-IM) indicates the total number of the correct responses for four trials in the WL-I, representing the short-term memory of the participants. The WL-Delayed Memory (WL-DM) indicates the number of the correct responses in the WL-II, representing the long-term memory of the participants. Higher scores of WL-IM or WL-DM represent better short-term or long-term memory.

The SCWT is a commonly used measure to assess inhibitory executive function. The SCWT is a sensitive measure to differentiate individuals with and without cognitive decline [23]. The differences in performance on congruent and incongruent tests may indicate the response interference. We used the differences of time between the congruent and incongruent tests to measure the inhibitory executive function. Higher scores of SCWT indicate poor inhibitory executive function.

### Statistical analysis

Skewness and kurtosis were measured, and all values were between 1 and − 1, which suggests the normal distribution of our data. Demographic variables and baseline characteristics were compared between the two groups using independent *t* tests and χ^2^ tests. The two-way mixed measures analysis of variance with group (HF vs. LF) factor as a between-subject factor and time (pre-training vs. post-training) as a within-subject factor, with Bonferroni correction, were used to investigate the effects of the two treatments on all outcomes. The effect size of partial eta squared (η^2^) was calculated to determine the magnitude of interaction and main effects for all outcomes, with an η^2^ of 0.14 for a large effect, 0.06 for a moderate effect, and 0.01 for a small effect. We used the paired *t* test as the post hoc analysis to evaluate directional changes from pre-training to post-training within groups. The statistical significance level was set at 0.05. Data were analyzed with PASW Statistics 18.0 software (IBM Corp., Armonk, NY, USA).

## Results

**Fig. 2:**
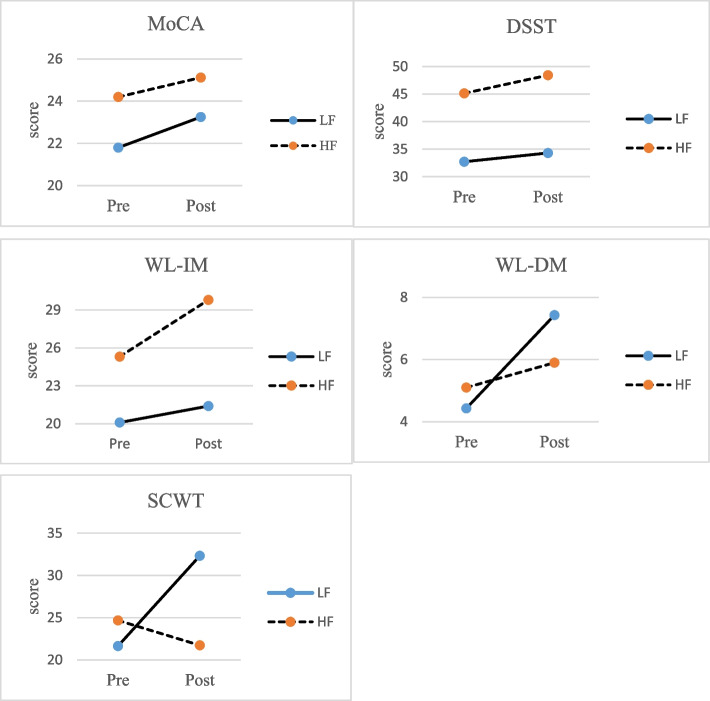
Line chart shows the mean scores of all outcomes at pre- and post-training. Abbreviations: MoCA, Montreal Cognitive Assessment; DSST, Digit Symbol Substitution Test; WL-IM, Word List-Immediate memory from Wechsler Memory Test; WL-DM, Word List-Delayed memory from Wechsler Memory Test; SCWT, Stroop Color Word Test; HF, high frequency; LF, low frequency

The study recruited 89 elderly people. The demographic data and baseline characteristics of two groups are listed in Table 1. The LF and HF groups were similar with respect to age, education, sex proportion, and Mini-Mental State Examination (MMSE) score (all *P* > 0.05) (see Table 1). Figure 2 shows the mean scores of all outcomes at pre- and post-training.

**Table 1:** Demographic data and baseline characteristics of the participants

Variables	Low Frequency	High Frequency	*P* Value
	(*n* = 40)	(*n* = 49)	
Demographic parameters			
Female sex, n (%)	25 (62.5)	40 (81.6)	0.06
Age, years	75.91 (8.75)	72.94 (8.2)	0.10
Education, years	8.58 (4.12)	10.06 (4.61)	0.12
MMSE score	24.83 (3.05)	25.88 (2.82)	0.10

*Abbreviations*: *MMSE* Mini-Mental State Examination

Data are presented as mean (standard deviation), unless indicated otherwise

There were significant group-by-time interaction effects on the WL-IM (*F*_(1,87)_ = 8.7, *P* = 0.004), WL-DM (*F*_(1,87)_ = 9.62, *P* = 0.003), and SCWT (*F*_(1,87)_ = 5.89, *P* = 0.017) scores (see Table 2). The post hoc tests showed significant improvement from pre-training to post-training in the HF group for MoCA (*t* =  − 2.95, *P* = 0.005), DSST (*t* =  − 3.49, *P* = 0.001), and WL-IM (*t* =  − 6.29, *P* < 0.001) and in the LF group for MoCA (*t* =  − 4.07, *P* < 0.001) and WL-DM (*t* =  − 5.81, *P* < 0.001).

**Table 2:** Descriptive and inferential statistics for the outcome measures

Outcome Measure	Low Frequency (*n* = 40)		High Frequency (*n* = 49)			Mixed ANOVA			
	Pre-training	Post-training	Pre-training	Post-training	Group	Time	Group by Time		
	Mean (SD)	Mean (SD)	Mean (SD)	Mean (SD)	*P*				η^2^
MoCA	21.80 (4.32)	23.25 (4.06)^a^	24.20 (4.7)	25.12 (4.63)^a^	0.023	< 0.001		0.262	0.014
DSST	32.72 (15.83)	34.31 (15.65)	45.14 (21.94)	48.43 (22.43)^a^	0.002	0.001		0.224	0.017
WL-IM	20.10 (7.27)	21.4(7.59)	25.31 (8.65)	29.8 (9.68)^a^	< 0.001	< 0.001		0.004	0.091
WL-DM	4.43 (3.13)	7.43 (3.24)^a^	5.1 (3.7)	5.9 (3.47)	0.504	< 0.001		0.003	0.100
SCWT	21.64 (10.03)	32.33 (25.88)	24.68 (16.10)	21.73 (14.23)	0.294	0.172		0.017	0.067

*Abbreviations*: *ANOVA* analysis of variance, *MoCA* Montreal Cognitive Assessment, *DSST* Digit Symbol Substitution Test, *WL-IM* Word List-Immediate memory from Wechsler Memory Test, *WL-DM* Word List-Delayed memory from Wechsler Memory Test, *SCWT* Stroop Color Word Test, *HF* high frequency, *LF* low frequency, *SD* standard deviation

^a^ < 0.05, when the score was greater than the pre-training score

Significant main effects of group were found for all outcome measures except the WL-DM (*F*_(1,87)_ = 0.45, *P* = 0.504) and SCWT (*F*_(1,87)_ = 1.12, *P* = 0.294). Significant main effects of time were found in all outcomes except the SCWT (*F*_(1,87)_ = 1.9, *P* = 0.172) (Table 2). The SCWT changes were in the opposite direction between the HF group (Δ =  − 2.95) and the LF group (Δ = 10.69). Moreover, WL-DM changes were primarily seen in the LF group (*t* =  − 5.81, *P* < 0.001) but not the HF group (*t* =  − 1.64, *P* = 0.107). Both groups of participants showed similar degrees of improvements in the MoCA (HF: *t* =  − 2.95, *P* = 0.005; LF: *t* =  − 4.07, *P* < 0.001) and DSST (HF: *t* =  − 3.49, *P* = 0.001; LF: *t* =  − 1.58, *P* = 0.122) from pre-training to post-training.

## Discussion

To our knowledge, this study is the first prospective study to directly compare the effects of combined physical and cognitive training in different frequencies on cognitive function for older adults with cognitive decline. We identified that the different training frequency of combined physical and cognitive training may result in benefits on different cognitive functions in older adults with cognitive decline. These findings are consistent with our hypothesis. The HF group demonstrated greater improvement in immediate memory measured by the WL-IM and in executive function measured by the SCWT than the LF group. Compared with the HF group, the LF group showed a great improvement in delayed memory measured by the WL-DM. Processing speed and global cognitive function increased in both HF and LF groups.

The results of this study indicate that improvements in immediate memory were greater after participants received combined training at a high training frequency than after a low training frequency. Immediate memory, a type of short-term memory, is the ability to remember information that has just been presented [24]. It allows us to hold on to information for a few minutes while we process it [24]. Repeatedly exposing the brain to new information and challenges through high-frequency training may strengthen the connections between neurons in the brain, improve an individual’s ability to remember and process information quickly, and result in good performance of short-term memory.

Our results are consistent with those of a previous study indicating that high-frequency training may lead to greater improvement of immediate (short-term) memory than long-term memory [16]. The findings suggested if short-term memory improvement is the intervention goal, the high-frequency training might be a necessary strategy [25]. Furthermore, the HF group in our study had superior performance in executive function compared with the LF group, similar to the results of the Bamidis et al. [26] study. Bamidis et al. found that the improvement of executive function was marginally significantly predicted by the larger number of training sessions. The possible reason may be that executive function, a higher-level cognitive function, may need more repetitive practice to learn the attentional control, working memory, inhibition, and problem solving.

The results showed that participants who received combined training at the low-training frequency had better performance in delayed memory (long-term memory) than those who received high-training frequency. It may be because practicing the new skill or activity at a low frequency may give the brain more time to process and consolidate the information that has been learned. Furthermore, the process of long-term memory may take more time, and rehearsing the information could help strengthen the memory trace and improve retention. Consistent with the previous studies, the low-frequency training could allow the participants to have more time to rehearse the skills or strategies they learned and elicit retrieval and reactivation of a memory trace, leading to better memory consolidation and retention [27, 28].

The HF and LF groups both had improvements in processing speed performance and global cognitive function. Global cognitive performance is usually used to screen the cognitive impairments and as the target of an intervention. Processing speed is one of the strongest predictors of age-related cognitive decline and the incidence of dementia [29, 30]. Processing speed is targeted ability of the training to reverse cognitive decline for the elderly [31] and is usually improved when cognitive training with a different frequency is applied [32, 33]. Accordingly, even one weekly session could result in benefits on processing speed ability and global cognitive function.

This study may provide evidence and guidance to practitioners that combined physical and cognitive training offering high or low frequency had positive effects on cognitive function for the elderly with cognitive decline. If environmental or personal reasons prevent the elderly with cognitive decline from going out frequently for the training sessions, one training session per week could also enhance cognitive function. Furthermore, different training frequency of combined physical and cognitive training may benefit different cognitive functions for the elderly with cognitive decline. Clinical practitioners could use different frequency training for distinctive intervention purposes. For example, for improving executive function, clinical practitioners could conduct combined physical and cognitive training at a high training frequency.

The current study has some limitations. First, although demographic data were similar between the HF and LF groups, the HF group showed better performance of global cognitive function, processing speed, and memory than the LF group at baseline. Thus, there may have been less room for improvement in the HF group after the combined physical and cognitive training. Future studies could adopt a stratified sampling method to avoid or reduce the bias.

Second, our results may not be applicable to older adults with a diagnosis of dementia and normal cognitive function. Future studies are needed to recruit participants with different cognitive levels, such as mild dementia or normal cognition, to verify these training effects on the elderly.

Third, lack of random assignment of participants to the intervention may have led to differences in baseline characteristics between two groups and affected the results. However, there were no differences in sex, age, educational levels, and the MMSE baseline score between two groups. The demographic and clinical characteristics might not bias the results, and no adjustments for the baseline characteristics were made.

Finally, the lack of a nontreatment control group may be considered a limitation. In the absence of a nontreatment control group, we could not control for learning effects that may influence post-training scores. Future studies should include a nontreatment control group that could control for these biases.

## Conclusion

This is the first prospective study to directly compare the effects of different training frequencies of combined physical and cognitive training on cognitive function for older adults with cognitive decline. Our study indicated that combined physical and cognitive training at different training frequencies may benefit different cognitive functions in older adults with cognitive decline. High-frequency training may lead to greater improvements in short-term memory and executive function, whereas low-frequency training may be more effective for improving long-term memory. These findings may assist clinical practitioners in choosing appropriate training frequencies based on various intervention purposes for the elderly with cognitive decline.

## Data Availability

The data sets used and/or analyzed during the current study are available from the corresponding author on reasonable request.

## Abbreviations

DSST: Digit Symbol Substitution Test
HF group: High training frequency
LF group: Low training frequency
MMSE: Mini-Mental State Examination
MoCA: Montreal Cognitive Assessment
SCWT: Stroop Color Word Test
WAIS: Wechsler Adult Intelligence Scale
WL: Word List
WL-I: The first part of Word List
WL-II: The second part of Word List
WL-DM: Word List -delayed memory
WL-IM: Word List -immediate memory
WMS-III: Wechsler Memory Scale—third edition
